# The Promise of Long-acting Injectable Antipsychotics for the Treatment of Bipolar I Disorder: The Role of Manic Predominance

**DOI:** 10.62641/aep.v53i5.1976

**Published:** 2025-10-05

**Authors:** Daniele Cavaleri, Ilaria Riboldi, Francesco Bartoli

**Affiliations:** ^1^School of Medicine and Surgery, University of Milano-Bicocca, 20900 Monza, Italy

Clinical research on the role of long-acting injectable (LAI) antipsychotics in the treatment of bipolar disorder (BD) is essential. The early use of LAIs is currently being investigated as a strategy to improve outcomes of BD [1, 2, 3]. Different factors beyond symptom severity, adherence, and relapse prevention must be taken into account when considering the use of LAIs to treat BD, especially in the earlier stages of the disease. Among these, the identification of the predominant polarity stands as crucial.

The concept of predominant polarity, since its introduction by Jules Angst in the late Seventies [4], is indeed gaining recognition as a valuable course specifier of BD [5, 6, 7]. According to the individual predisposition to mood relapses over the course of the illness, subjects with BD can be divided into three subgroups: (1) those with “manic predominance”, characterized by a higher frequency of manic episodes; (2) those with “depressive predominance”, who suffer mostly from depressive episodes; and (3) the “nuclear type”, with a balanced proportion between manic and depressive episodes. Considering the currently most common definition [7], individuals with a manic-to-depressive episodes ratio >2/3 are considered to have a manic predominant polarity, while those with a ratio <1/3 are considered to have a depressive predominant polarity. Meta-analytic data show similar rates for these conditions, each affecting around one third of individuals with BD [8]. However, some geographical differences were observed [8], with higher rates of manic predominance in South America (Brazil and Colombia) and Asia (India and Singapore) compared to Europe, although data in most countries is not yet available (Fig. 1).

**Fig. 1.**
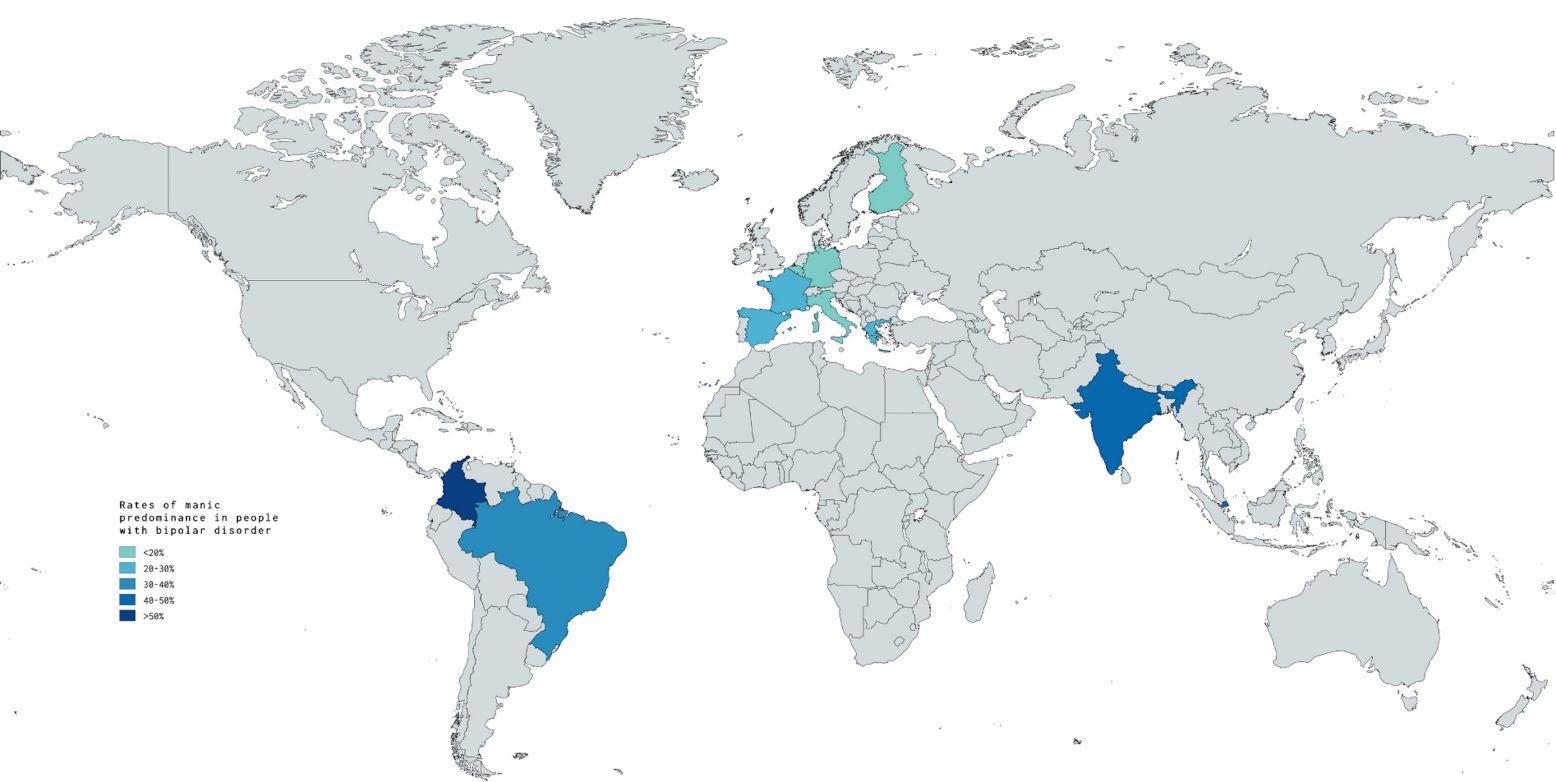
**Rates of manic predominance in people with bipolar disorder worldwide**. Created with MapChart (https://www.mapchart.net/).

Moreover, in addition to these disorders with a bipolar course, the affective spectrum also includes “unipolar mania”, a condition characterized by recurrent manic episodes without any history of depressive ones [9]. Nosologically counterposed to unipolar depression, unipolar mania is not as rare as previously thought. Recent estimates suggest that up to 10% of subjects diagnosed with BD type I in Europe have never experienced depressive episodes [10], although rates in other countries might be significantly higher [11].

The predisposition to experience a greater number of manic episodes — or even manic episodes only — is likely influenced by many different clinical and environmental factors. Features such as younger age, male gender, an earlier and manic onset, a diagnosis of BD type I, and the presence of psychotic features have been associated with both a manic predominant polarity and unipolar mania [8, 9], while a depressive onset, the number of mood episodes, and a history of suicide attempts have been associated with a depressive predominant polarity [8].

These distinct profiles highlight that certain subsets of individuals with BD displaying specific clinical features may have differential therapeutic needs. Therefore, overcoming the often misleading distinction between type I and type II [12] by focusing on the predominant polarity may provide a more accurate and clinically relevant approach in real-world settings. Indeed, by differentiating subjects with BD based on their predisposition towards mania or depression, it may be possible to uncover new insights for clinical practice.

Indeed, the utility of LAIs in BD is notably supported by evidence emerging from mirror-image studies [2]. LAI treatment seems to be associated with a significant reduction of manic relapses, while their effectiveness for depressive episodes is less clear. In addition, LAI treatment initiation was associated with a lower number of both hospital admissions and emergency department visits in the year after LAI initiation [2].

In view of this, individuals exhibiting a manic predominant polarity — and even more those with unipolar mania — may actually represent an ideal population to whom propose treatment with LAIs, given their effectiveness in preventing manic relapses and related psychotic features. This stands as especially true considering that these individuals are likely to be less adherent to psychopharmacological treatment [13]. Even though the primary indication of second-generation LAIs — such as aripiprazole monohydrate, olanzapine pamoate, paliperidone palmitate, and risperidone microspheres or in situ microparticles — is schizophrenia, these drugs have a role in treating BD when a maintenance treatment with these agents is needed but adherence to oral treatments is poor.

Despite the potential of LAIs, significant barriers remain to their widespread adoption: first, their use for BD in European countries is not approved and remains off-label; second, clinicians often show and attitudinal resistance towards their prescription (especially in the early stages of the disease); third, their effect in the prevention, or on potential induction, of depressive episodes in BD is still uncertain. Regarding this latter issue, it is likely that the putative depressogenic effect of antipsychotics may depend on the subject’s polarity proneness. This once again highlights the importance of routinely assessing the patient’s predominant polarity before starting a LAI for treating or preventing mania in BD.

Nonetheless, the awareness of the possible utility of LAIs for BD should not overshadow the role of lithium, that remains the gold standard for the treatment of BD, especially in subjects prone to manic recurrencies [14], including those with unipolar mania. As one of the few available drugs in psychiatry with neuroprotective and disease-modifying effects [15], it is essential to prioritize psychoeducational and behavioral interventions to improve medication adherence and facilitate the prescription of lithium, which should still be considered the primary option, while LAIs should be used as an add-on or second-line treatment for BD with a manic predominance.

As we move toward personalized psychiatry, the integration of the concept of predominant polarity into the management framework of BD stands as essential for optimizing its treatment. With this in mind, clinicians can gain additional insights into the beneficial or deleterious effects that available treatments may have on the natural course of the illness. It is important to unlock the full potential of LAIs in treating BD by identifying those subjects who may benefit them the most: among these, people characterized by a manic predominant polarity and those with unipolar mania should be considered primary candidates. Given the lack of robust guidelines incorporating these concepts, the centrality of LAI use in people with BD must be acknowledged and endorsed. Nothing more than keeping the predominant polarity in mind can make us clinicians aware of the possible role of LAIs in the treatment of BD.

## Availability of Data and Materials

Not applicable.
